# GBM Derived Gangliosides Induce T Cell Apoptosis through Activation of the Caspase Cascade Involving Both the Extrinsic and the Intrinsic Pathway

**DOI:** 10.1371/journal.pone.0134425

**Published:** 2015-07-30

**Authors:** Barun Mahata, Soumika Biswas, Patricia Rayman, Ali Chahlavi, Jennifer Ko, Ashish Bhattacharjee, Yu-Teh Li, Yuntao Li, Tanya Das, Gaurisankar Sa, Baisakhi Raychaudhuri, Michael A. Vogelbaum, Charles Tannenbaum, James H. Finke, Kaushik Biswas

**Affiliations:** 1 Division of Molecular Medicine, Bose Institute, Kolkata, India; 2 Department of Immunology, Lerner Research Institute, Cleveland Clinic, Cleveland, United States of America; 3 Spine and Brain Institute, St. Vincent Medical Center, Jacksonville, Florida, United States of America; 4 Pathology Institute, Cleveland Clinic, Cleveland, United States of America; 5 Department of Biotechnology, National Institute of Technology, Durgapur, India; 6 Department of Biochemistry and Molecular Biology, Tulane University School of Medicine, New Orleans, United States of America; 7 Brain Tumor and Neuro-oncology Center in the Neurological Institute, Cleveland Clinic, Cleveland, United States of America; University of Pittsburgh, UNITED STATES

## Abstract

Previously we demonstrated that human glioblastoma cell lines induce apoptosis in peripheral blood T cells through partial involvement of secreted gangliosides. Here we show that GBM-derived gangliosides induce apoptosis through involvement of the TNF receptor and activation of the caspase cascade. Culturing T lymphocytes with GBM cell line derived gangliosides (10-20μg/ml) demonstrated increased ROS production as early as 18 hrs as indicated by increased uptake of the dye H_2_DCFDA while western blotting demonstrated mitochondrial damage as evident by cleavage of Bid to t-Bid and by the release of cytochrome-c into the cytosol. Within 48-72 hrs apoptosis was evident by nuclear blebbing, trypan blue positivity and annexinV/7AAD staining. GBM-ganglioside induced activation of the effector caspase-3 along with both initiator caspases (-9 and -8) in T cells while both the caspase-8 and -9 inhibitors were equally effective in blocking apoptosis (60% protection) confirming the role of caspases in the apoptotic process. Ganglioside-induced T cell apoptosis did not involve production of TNF-α since anti-human TNFα antibody was unable to protect T cells from nuclear blebbing and subsequent cell death. However, confocal microscopy demonstrated co-localization of GM2 ganglioside with the TNF receptor and co-immunoprecipitation experiments showed recruitment of death domains FADD and TRADD with the TNF receptor post ganglioside treatment, suggesting direct interaction of gangliosides with the TNF receptor. Further confirmation of the interaction between GM2 and TNFR1 was obtained from confocal microscopy data with wild type and TNFR1 KO (TALEN mediated) Jurkat cells, which clearly demonstrated co-localization of GM2 and TNFR1 in the wild type cells but not in the TNFR1 KO clones. Thus, GBM-ganglioside can mediate T cell apoptosis by interacting with the TNF receptor followed by activation of both the extrinsic and the intrinsic pathway of caspases.

## Introduction

A feature of many tumors is their ability to evade detection and destruction by the host immune system [1, 2] including glioblastoma multiforme (GBM) which is most proficient in this regard [3, 4]. Though GBM develops and remain primarily within the brain, it can still induce local and systemic host immunosuppression [5, 6]. Several mechanisms have been proposed for the observed immune suppression, including locally secreted factors (TGF-β and IL-10) [1, 7–11] along with the action of regulatory T cells (Tregs) and myeloid derived suppressor cells (MDSCs) [12–15]. Furthermore, previous studies on mechanisms by which tumor cells induce T cell apoptosis implicated tumor associated Fas ligand (FasL) and other tumor necrosis factor (TNF)-related ligands in the process [16, 17]. Similar dysfunction of the immune system is observed when tumor cell conditioned medium is added to human T cells. Additionally, tumor cyst fluids and cerebrospinal fluids from patients with gliomas are known to be immunosuppressive [18]. These *in vitro* findings are consistent with the observation that compared to healthy donor T cells a portion of peripheral blood T cells from GBM patients [19] or T cells infiltrating GBM [20] are apoptotic, indicating that glioma mediated immune-suppression may be caused in part by soluble mediators.

Tumors have been known to overexpress various gangliosides [21–25] with varying immunosuppressive potential. Gangliosides have been found to inhibit multiple steps in the cellular immune responses including antigen processing and presentation [26], T-cell proliferation [27] and production of cytokines, such as IL-1β and IFN-γ [28]. In fact, reports from our laboratory and others have demonstrated gangliosides as one of the soluble mediators of tumor induced T cell apoptosis [29–31]. Although various studies have described the role of gangliosides in mediating apoptosis of different immune cells [22, 29], there is minimal data demonstrating the precise mechanistic pathways through which tumor derived gangliosides mediate T lymphocyte death.

Here we describe the mechanism by which GBM cell line isolated gangliosides mediate T cell apoptosis. This process involves the activation of the caspase cascade through both receptor dependent (extrinsic) and receptor independent (intrinsic) pathways. Data further shows that GBM derived gangliosides recruit death domains (TRADD and FADD) through its direct interaction with the TNF receptor-I (TNF-RI), that is independent of TNF ligand in GBM ganglioside mediated T cell apoptosis.

## Materials and Methods

### Reagents

Anti-human CD41 tetramer and human T cell enrichment cocktail were obtained from StemCell Technologies, Vancouver, Canada. Standard gangliosides were purchased from Matreya, Pleasant Gap, PA. Hamster monoclonal anti-GM2 antibody (DMF10.167.4) was a gift from Dr. Kenneth Rock, Department of Pathology, University of Massachusetts Medical School, Worcester, MA [32] while anti-human GD1a antibody was purchased from Seikagaku Corporation, Tokyo, Japan [33]. Peroxidase conjugated goat anti-hamster IgG and rabbit anti-mouse IgM were obtained from Jackson ImmunoResearch, West Grove, PA. AlexaFluor 488 goat anti-hamster IgG and CM-H_2_DCFDA were purchased from Invitrogen, Eugene, OR. Recombinant human TNF-α, anti-armenian hamster IgG, and anti-mouse IgM antibodies were purchased from Santa Cruz Biotechnology, Santa Cruz, CA while goat anti-human cytochrome-c was purchased from BD Pharmingen, San Jose, CA. Paraformaldehyde was purchased from Electron Microscopy Sciences, Hatfield, PA, USA. Vectashield mounting media containing 4’-6’-diamidino-2-phenylindole (DAPI) was obtained from Vector Laboratories, Inc., Burlingame, CA. AnnexinV-PE and 7-AAD were obtained from BD Biosciences, San Jose, CA. HPLC grade methanol and analytical grade chloroform, isopropanol, diisopropyl ether and n-butanol were obtained from Fisher Scientific, Fair Lawn, NJ. DiOC_6_ was obtained from Molecular Probes, Eugene, OR. Fluorochrome Inhibitors of Caspases (FLICA) for immunoflowcytometric staining of caspases 3, 8 and 9 were purchased from Immunochemistry Technologies, Bloomington, MN. Precoated LHPKD silica gel 60Å high performance thin layer chromatographic (HPTLC) plates were obtained from Whatman, Inc., Clifton, NJ. Rabbit anti-human cleaved caspase 3 and 8, t-Bid, TRADD, FADD and TNF-RI antibodies for western were obtained from Cell Signaling Technologies, Danvers, MA. Mouse anti-human TNFRI antibody for immunoprecipitation and confocal microscopic studies was purchased from Abcam (# ab2257), Cambridge, MA and TNFR1 antibody for western blotting was purchased from Cell Signaling Technology (#3736), USA. Mouse anti-human TNFα antibody and human apoptosis proteome profiler kit were purchased from R & D systems, Minneapolis, MN. Inhibitors to Caspase-3 (Z-DEVD-FMK), 8 (Z-IETD-FMK), 9 (Z-LEHD-FMK) and Pan caspase (Z-VAD-FMK) inhibitors were ordered from Medical & Biological Laboratories Co., Ltd., Woburn, MA. Complete media (RPMI 1640, Cleveland Clinic Media Core) consists of 10% fetal bovine serum (FBS) (Hyclone, Logan, UT), 2mM L-glutamine, 50μg/liter gentamicin, 100mM MEM sodium pyruvate and 10mM MEM non essential amino acids (Invitrogen, Carlsbad, CA). TNFR1 TALEN plasmids were a kind gift from Dr. Jin-Soo Kim of the National Creative Research Initiatives Center for Genome Engineering and Department of Chemistry, Seoul National University, Seoul, South Korea [34] Amaxa nucleofector Kit V was purchased from Lonza, Germany. G418 was obtained from Hi-Media, Mumbai, India.

### Cell Lines

GBM cell lines CCF52, CCF4 and U87 [29] were obtained from Dr. Vogelbaum (Cleveland Clinic Foundation, Cleveland, OH) and maintained in complete RPMI 1640 medium at 37°C with 5% CO_2_. Jurkat T cells and the TNFR1 KO (knockout) clones were maintained in complete RPMI under usual cell culture conditions.

### Isolation of peripheral blood T lymphocytes from normal donors

Peripheral blood mononuclear cells (PBMCs) were isolated from normal healthy donors with informed written consent in accordance with the guidelines of the Cleveland Clinic Institutional Review Board (IRB 4639), which approved this study. Briefly, PBMCs were isolated by Ficoll-Hypaque (Amersham Pharmacia Biotech AB, Uppsala, Sweden) density gradient centrifugation [22, 35]. T cells were isolated (97% CD3+) by negative magnetic selection using microbeads coated with antibodies to macrophages, NK cells, B cells and RBCs (Stem Cell Technologies). Primed T cells were also used in some of the experiments, which were generated by stimulating T cells (1×10^6^/ml) in flasks precoated with 10μg/ml anti-CD3 and 5μg/ml anti-CD28 Abs for 3 days followed by expansion for 7–10 days in the presence of 200 IU/ml IL-2.

### Ganglioside isolation from GBM cell lines

Ganglioside extraction was done using chloroform: methanol (1:1) for 18hrs at 4°C followed by partitioning in 10ml of diisopropyl ether/1% butanol/0.1% aqueous NaCl as described previously [22, 36]. Finally, isolated gangliosides were further purified by passing through a Sephadex G-25 column to get rid of contaminating salts and other small molecular weight impurities and stored under nitrogen at -20°C freezer.

### HPTLC and ELISA analysis of isolated gangliosides

HPTLC analysis of isolated gangliosides was done using precoated 10×10cm silica gel 60Å HPTLC glass plates as previously described [22]. ELISA was performed both to determine specificity of the anti-ganglioside antibodies and to assess the composition of the isolated GBM gangliosides as previously described [33, 37]. Briefly, CCF52 gangliosides were coated on a 96 well flat bottomed ELISA plate and immunostained with 1μg/ml of both hamster human GM2 Ab (DMF10.167.4) or anti-human GD1a Ab (Seika Gaku) followed by staining with HRP-conjugated goat anti-hamster IgG or rabbit anti-mouse IgM, respectively.

### Immunofluorescent Microscopic Analysis

T cells were treated with or without GBM derived gangliosides, followed by attaching the cells on glass slides precoated with poly-L-Lysine for 1hour at 37°C. Cells were then fixed with 3.7% paraformaldehyde (PFA) for 10 min., washed with 1X PBS, following which, cells were mounted with Vectashield mounting medium containing 4’, 6-diamidino-2-phenyindole (DAPI) to visualize the nuclei. A total of 200 cells were counted to assess the percentage of T cells showing nuclear blebbing.

For co-localization studies, T cells treated or not with CCF52 gangliosides, or Jurkat clones treated with or without ganglioside GM2 were placed on poly-L-Lysine coated coverslips, fixed with 3.7% PFA as described previously. Cells were then blocked with 1% BSA for 30min at room temperature followed by incubation with hamster anti-human GM2 Ab (1:100) and mouse anti-human TNFR1 Ab (1:50) for 2h, and then washed with 1X PBS. Cells were incubated with appropriately labeled 2°Abs (Alexa Fluor 488 goat anti-hamster IgG and Alexa Fluor 594 donkey anti-mouse IgG). After washing with 1X PBS, cells were mounted with Vectashield mounting medium containing DAPI to visualize the nuclei.

### Immunocytometric analysis of T cells for Apoptosis

T cells isolated from normal donors were cultured with or without GBM gangliosides for 48-72hrs. Thereafter, lymphocytes were suspended in 1X annexinV binding buffer and stained with annexinV-PE and 7-AAD for 15min at room temperature. A minimum of ten thousand events were acquired on a FACS-Calibur multivariable flow cytometer and analyzed using CellQuest version 3.3 software.

An aliquot of the T cells, following culture, was examined for viability by trypan blue exclusion method as described earlier [22].

### Analysis of reactive oxygen species (ROS) and caspase activation in T cells

T lymphocytes were incubated in 6-well plates at 1×10^6^ cells/ml in the presence or absence of CCF52 gangliosides for 18, 48 or 72hrs at 20μg/ml and 2μM H_2_O_2_ for 18hrs, which was used as a positive control (data not shown). Cells were then stained for active caspase 3, 8 and 9, ROS, annexinV and 7-AAD. To study activation of caspases, cells were stained with fluorochrome inhibitors of caspases (FLICA), Immunochemistry Technologies. Induction of ROS was determined by staining the cells with CM-H_2_DCFDA (Invitrogen) for 4h at 37°C. Induction of apoptosis was also examined by surface staining with annexinV-PE and 7-AAD.

### Cell Lysates and analysis for Western Blot

Western immune-blot was done according to methods described earlier [38]. The immune-reactive proteins were visualized using HRP conjugated secondary antibodies and enhanced chemiluminescence reagent (ECL Western Blotting Kit, Amersham).

### Cytochrome c Assay

Following culture of T cells with GBM derived gangliosides, cells were washed with 1X PBS, and once with mitochondria isolation buffer [20mM HEPES-KOH, 10mM KCl, 1.5mM MgCl_2_, 1mM EGTA, and 250mM sucrose, containing protease inhibitor cocktail] [38]. Cell pellet was resuspended in 100μl of mitochondria isolation buffer, followed by homogenization using a Dounce Homogenizer. The homogenate was centrifuged at 750×g for 20min, and the supernatant, containing released cytochrome c, was assessed for protein concentration. Equivalent amounts of protein were then resolved on 12% SDS-PAGE gels, and processed for western immune-blot analysis.

### Detection of mitochondrial permeability transition (MPT) in T cells

MPT in T cells cultured with GBM derived gangliosides was studied as described previously [22]. Reduced uptake of the dye, DiOC_6_ as observed under a fluorescent microscope, indicated activation of MPT.

### Human Apoptosis Array for Proteome Profiling

T cells were treated with or without CCF52 ganglioside (15μg/ml) for 48hrs. Thereafter, cell lysates were subjected to analysis using the Proteome Profiler human apoptosis antibody array kit from R&D Systems (Minneapolis, MN) according to the manufacturer’s instructions. Arrays were developed with streptavidin-HRP for 30min on a rocking platform shaker. Developed signals were densitized using Image J software, pixel densities were normalized to untreated sample and expressed as mean pixel density.

### Co-immunoprecipitation of TNFRI from T cells treated with GBM gangliosides to show downstream binding of FADD and TRADD

T cells (10×10^6^) were treated with CCF52 gangliosides (15μg/ml) for different times, lyzed and protein content was measured by usual method. Protein A beads were centrifuged at 2300rpm/500×g for 5min at room temperature and the supernatant was discarded. 500μl beads was washed with an equal volume of RIPA buffer, centrifuged, supernatant was discarded and the washing was repeated at least thrice. Finally, beads were resuspended in an equal volume of RIPA buffer. 250μg of protein lysates were incubated with mouse monoclonal anti-TNFRI antibody (6μg/condition) in a final volume of 500μl (with 1X PBS) in the cold room for 3-4hrs in a rocker. 50μl of washed protein A beads were added to each tube containing the antibody-lysate system and rocked in the cold room overnight. Protein A beads were centrifuged (13,000rpm or maximum speed) at 4°C for 1min and washed at least 5 times using RIPA buffer as described before. Finally, beads were resuspended in 35μl of 2X loading buffer, boiled in water bath for 10min, centrifuged for 5min at maximum speed, and the supernanant was loaded on a SDS-PAGE 4–15% Tris-HCl gradient gel (BioRad). Western immunoblot was done for FADD, TRADD and TNFRI as described in previous section.

### TALEN mediated knockout and generation of stable TNFR1 knockout (KO) Jurkat clones

1×10^6^ Jurkat-T cells were transfected with 1μg each of TNFR1 specific TALEN pairs along with 25ng of pD2-EGFP-N1 (40:40:1) [39] using Amaxa nucleofector IIb and transfection kit V according to manufacturer’s protocol. Following recovery post 48hrs of transfection, cells were selected against 1μg/ml G418 for another 15 days. Thereafter cells were expanded and TNFR1 expression was checked by western blot analysis. G418 selected TNFR1 transfected pool of Jurkat-T cells were selected for further experimentation, since they showed negligible TNFR1 expression.

### Statistical Analysis

Statistical analysis was performed by Student's t-test using Graphpad Prism 5.0. Values were considered as statistically significant when p values were less than 0.05.

## Results

### GBM cell line derived gangliosides induce apoptosis of T lymphocytes

**Fig 1:**
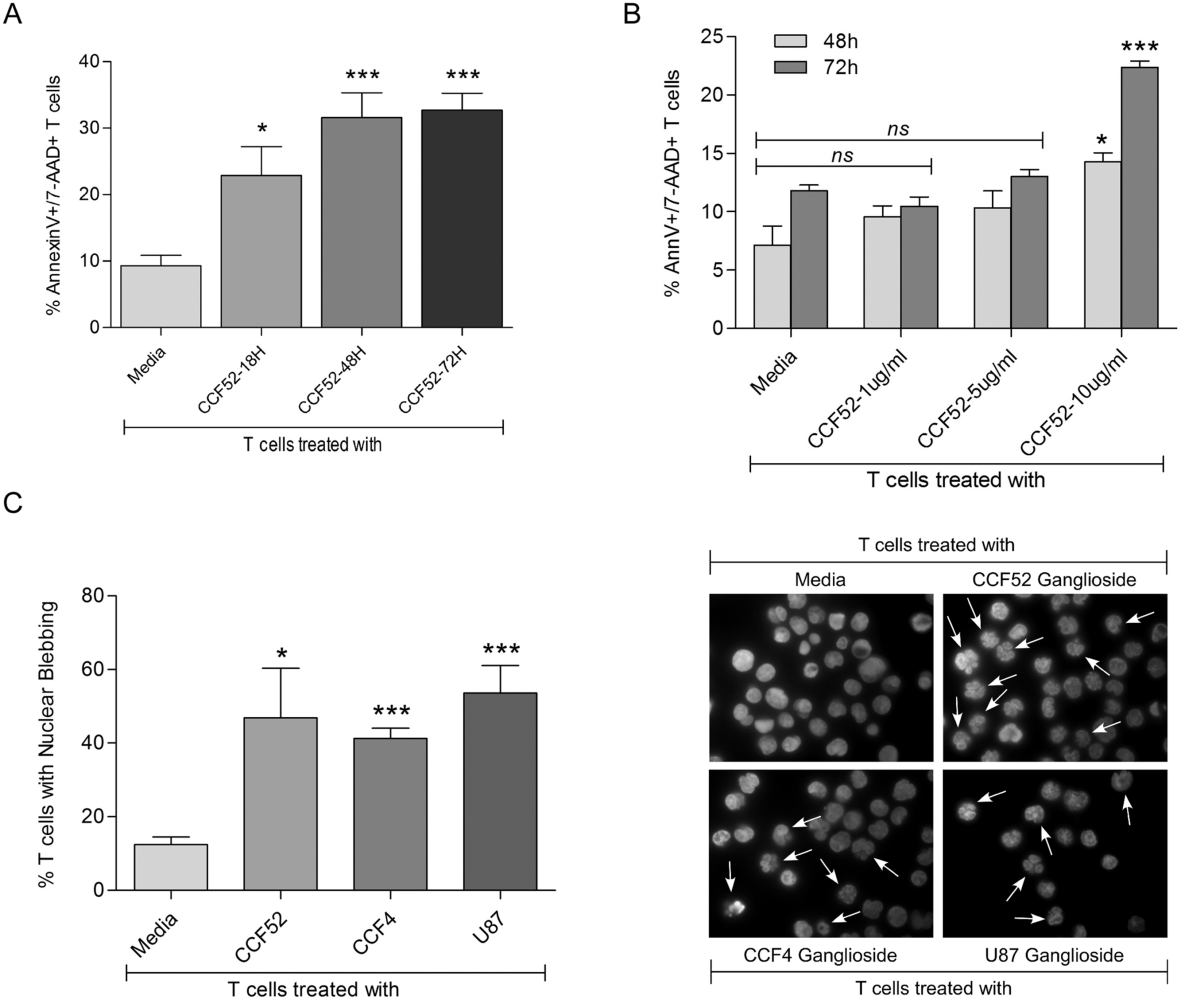
GBM derived gangliosides induce apoptosis of T cells. Peripheral blood T lymphocytes were isolated and purified from blood of healthy volunteers by negative selection. T lymphocytes were co-cultured with 15μg/ml of CCF52 derived gangliosides for 18–72 hrs, followed by annexinV-PE/7AAD staining for flow cytometric estimation of apoptosis, as shown in Fig 1A (*p<0.05 vs Media, ***p<0.001 vs Media). Purified T cells were incubated with different concentration (1–10μg/ml) of CCF52 gangliosides for 48hrs and 72hrs and apoptosis was measured by flow cytometric analysis using annexinV-PE/7AAD staining as indicated in Fig 1B (*p<0.05 vs Media, ***p<0.001 vs Media). Fig 1C shows graphical representation showing the percentage of nuclear blebbing event in T cells treated with gangliosides (15μg/ml) isolated from different glioblastoma cell lines for 72hrs (*p<0.05 vs Media, ***p<0.001 vs Media). Fig 1D shows representative photomicrograph demonstrating induction of apoptosis in T cells following 72hrs treatment with three different GBM derived gangliosides (15μg/ml) as evidenced by microscopic evaluation of nuclear blebbing by staining with DAPI. Data represents mean of at least 3 independent experiments unless mentioned otherwise.

T cells were treated with gangliosides (15μg/ml) isolated from the GBM cell line CCF52 at varying time intervals (Fig 1A) or with varying ganglioside concentration (Fig 1B), followed by staining with annexinV and 7-AAD prior to FACS analysis. CCF52 gangliosides induced time dependent T cell apoptosis, as evidenced by increased annexinV^+^/7-AAD^+^ staining at 18hrs (20%) and reaching peak at 72hrs (32%) (Fig 1A). However, significant T cell apoptosis was only observed at 10μg/ml or higher concentration as shown in Fig 1B. Although, CCF52 gangliosides did not show any significant induction in T cell apoptosis at lower concentrations, both GBM gangliosides CCF52 and CCF4 however, showed significant suppression of IFN-γ expression indicating inhibition of T cell effector function at concentrations as low as 1μg/ml as shown in S1 Fig. Cell membrane blebbing and nuclear condensation were used as an index of apoptotic cell morphology [40]. As seen in Fig 1C, fluorescent microscopy revealed membrane blebbing and nuclear condensation characteristic of ongoing apoptosis (Fig 1C) in T cells treated with GBM derived gangliosides isolated from three different tumor lines, CCF52, CCF4 and U87 at 72hrs. Fig 1D shows photomicrograph of a single field representing induction of nuclear blebbing in T cells exposed to GBM gangliosides.

### Induction of T cell apoptosis by GBM derived gangliosides is mediated through activation of caspases

**Fig 2:**
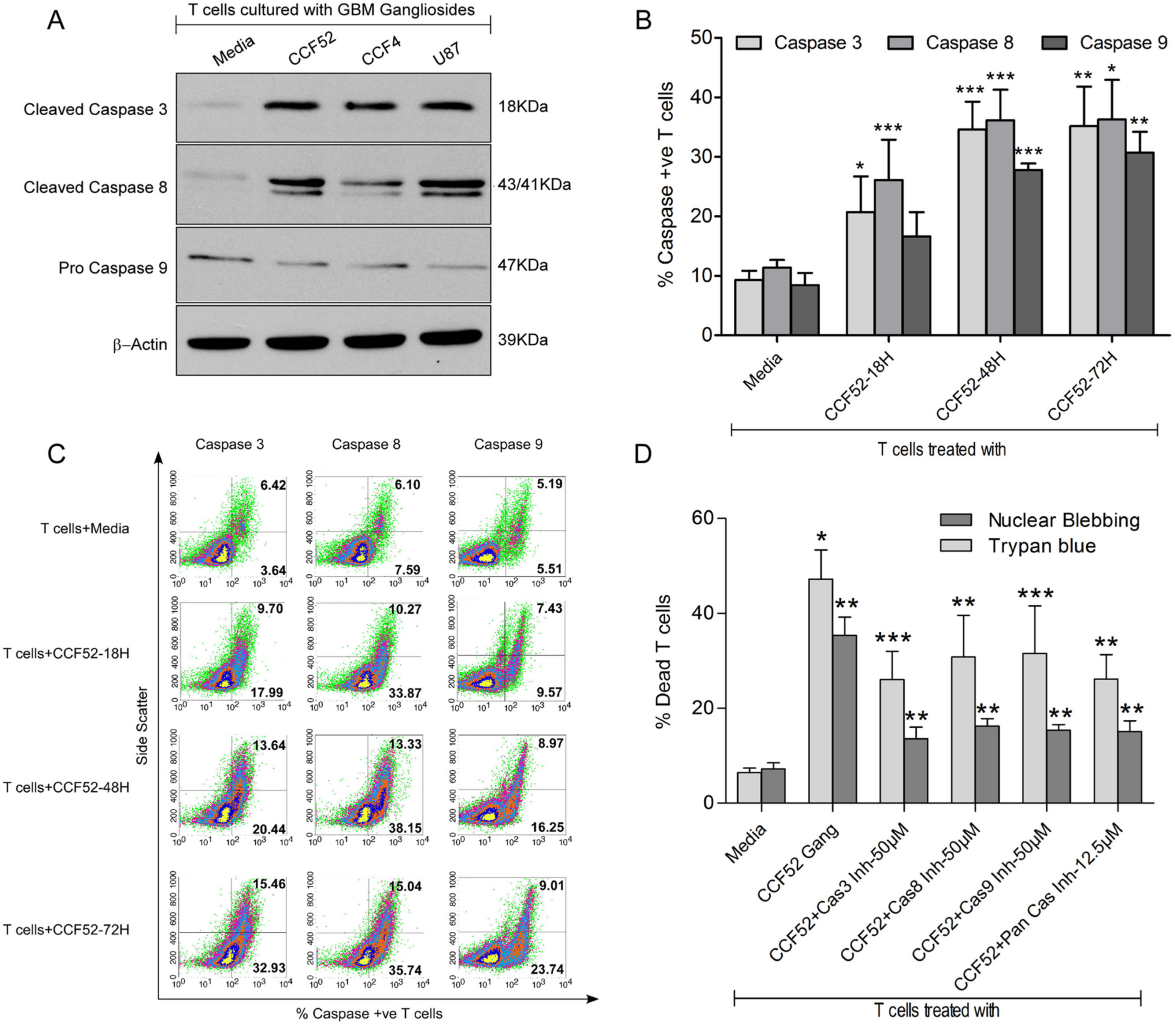
Induction of T cell apoptosis by GBM derived gangliosides is mediated through caspase activation. Lysates from T cells treated with GBM derived gangliosides for 72hrs were resolved in a 12% SDS-PAGE and western immunoblot was performed to detect expression of caspases, as shown in Fig 2A. Fig 2B shows graphical representation demonstrating time dependent induction of caspases in purified T cells in response to CCF52 gangliosides for 18hrs, 48hrs & 72hrs as measured by staining the cells with fluorochrome labeled inhibitors of caspases (FLICA) (*p<0.05 vs Media, **p<0.01 vs Media, ***p<0.001 vs Media). Cells were acquired on a FACS-calibur multivariable flow cytometer and % caspase +ve T cells were analyzed using CellQuest 3.3 software. Representative density plot showing time dependent induction of caspases in T cells is shown in Fig 2C. Pre-treatment of T cells with inhibitors of caspases-3, -8, -9 (at 50μM each) and a pan caspase inhibitor (at 12.5μM) 2hrs prior to ganglioside (15μg/ml) treatment, significantly protected T cells from CCF52 ganglioside induced T cell death as evident by trypan blue exclusion in Fig 2D (*p<0.05 vs Media; **p<0.01 vs CCF52 ganglioside-15μg/ml; ***p<0.001 vs CCF52 ganglioside-15μg/ml), and by microscopic analysis of nuclear blebbing in Fig 2D (**p<0.01 vs Media; **p<0.01 vs CCF52 ganglioside-15μg/ml) as shown in Fig 2D. Data represents mean of at least 3 independent experiments unless mentioned otherwise.

To understand the signaling events involved in ganglioside mediated T cell death, caspase activation was determined by immunoblotting using antibodies to caspase-3, caspase-8 and -9 following incubation of isolated T cells with gangliosides derived from 3 distinct GBM lines (72 hrs). Induction of the effector caspase-3 and caspase-8 (Fig 2A) was demonstrated by increased expression of the corresponding cleaved products while caspase-9 was also found to be activated as evident by decreased expression of its pro-form (Fig 2A). β-actin was used as the endogenous control with no change noted. The time frame (18, 48 and 72hrs) for activation of different caspases in T lymphocytes treated or not with CCF52 ganglioside was assessed by staining with fluorochrome labeled inhibitors of caspases (FLICA) followed by flow cytometric analysis as shown graphically in Fig 2B. Gangliosides induced activation of caspases-3, -8 and -9 by 18hrs, however, careful analysis of the representative density plot shows that although, caspase 8 activity reached a maximum at 48hrs, caspase-3 and -9 activity reached maximum at 72hrs (Fig 2C). Inhibitors specific to caspases-3 (Z-DEVD-FMK), -8 (Z-IETD-FMK) or -9 (Z-LEHD-FMK) and a pan caspase inhibitor (Z-VAD-FMK) when added to T cells 3hrs prior to addition of CCF52 ganglioside caused significant reduction of T cell death (~60%) when compared to that of untreated cells, as evidenced from trypan blue assay as well as nuclear blebbing, suggesting that ganglioside induced caspase activation and subsequent T cell apoptosis likely involves both receptor mediated (caspase-8) and mitochondrial (caspase-9) pathway of caspase activation (Fig 2D).

### GBM derived gangliosides induce ROS, cause mitochondrial damage and release cytochrome-c from the mitochondria

**Fig 3:**
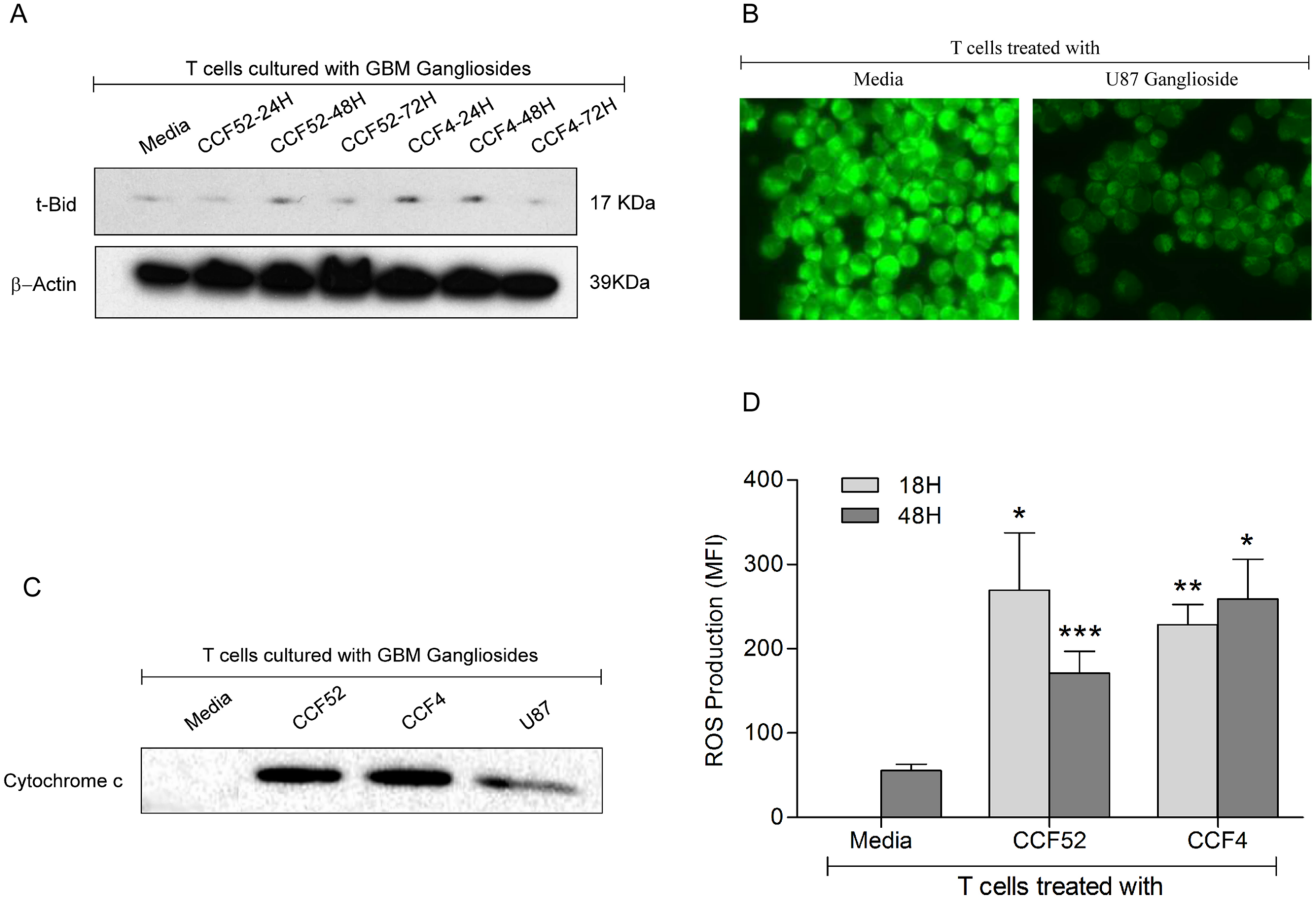
Involvement of mitochondria in GBM ganglioside mediated caspase activation. T cells were co-cultured with GBM derived gangliosides CCF52 and CCF4, and t-Bid induction was detected as shown in Fig 3A. Reduced fluorescence of DiOC_6_ in T cells treated with U87 gangliosides (15μg/ml) for 48hrs, versus control cells is indicative of mitochondrial damage (Fig 3B). Evidence of mitochondrial damage was also observed in T cells exposed to GBM derived gangliosides as evidenced from mitochondrial cytochrome c release (Fig 3C). Time dependent induction of reactive oxygen species (ROS) was measured in purified T cells treated with 15μg/ml CCF52 and CCF4 derived gangliosides for 18hrs and 48hrs, as compared to media control for 48hrs, evidenced by H_2_DCFDA staining and flow cytometric analysis (Fig 3D) (*p<0.05 vs Media, **p<0.01 vs Media, ***p<0.01 vs Media). ROS production is represented as the mean fluorescence intensity (MFI) of at least 3 independent experiments.

Data presented in the previous section of this manuscript demonstrated time dependent activation of caspase 8 (Fig 2). Since, apoptosis signals can be amplified through caspase-8 dependent release of cytochrome c from the mitochondria through formation of t-Bid [41] ultimately leading to caspase-9 activation, experiments were performed to see whether GBM derived gangliosides induce formation of t-Bid. Western blot analysis demonstrates time dependent induction of t-Bid (17KDa) in T cells treated with GBM gangliosides (Fig 3A). CCF52 mediated induction of t-Bid peaks at 48hrs, while CCF4 mediated t-Bid induction occurs much earlier at 24hrs (Fig 3A). Since, t-Bid induction is usually associated with a change in mitochondrial permeability potential (MPT), we tested whether GBM gangliosides caused disruption of MPT. As seen in Fig 3B, treatment of T cells with U87 ganglioside results in mitochondrial damage through an induction of MPT as observed by the decreased fluorescence of DiOC_6_ dye in U87 treated T cells versus the control (Fig 3B), indicating a change in the mitochondrial permeability. Evidence of mitochondrial damage was further supported by western blot analysis of mitochondrial lysates from T cells cultured with GBM gangliosides which shows release of cytochrome c from the mitochondria (Fig 3C) of T cells exposed to GBM derived gangliosides but not media. As observed in Fig 3D, incubation of T cells with both GBM derived gangliosides caused significant induction of ROS formation, which tripled with CCF52 or doubled with CCF4 ganglioside within 18hrs. Interestingly, however, although ROS formation kept on increasing thereafter with CCF4 ganglioside, it dropped significantly with CCF52 ganglioside by 48hrs (Fig 3D).

### Apoptosis proteome profiler array demonstrates increase of pro-apoptotic proteins and decrease of anti-apoptotic proteins in T cells in response to CCF52 ganglioside

**Fig 4:**
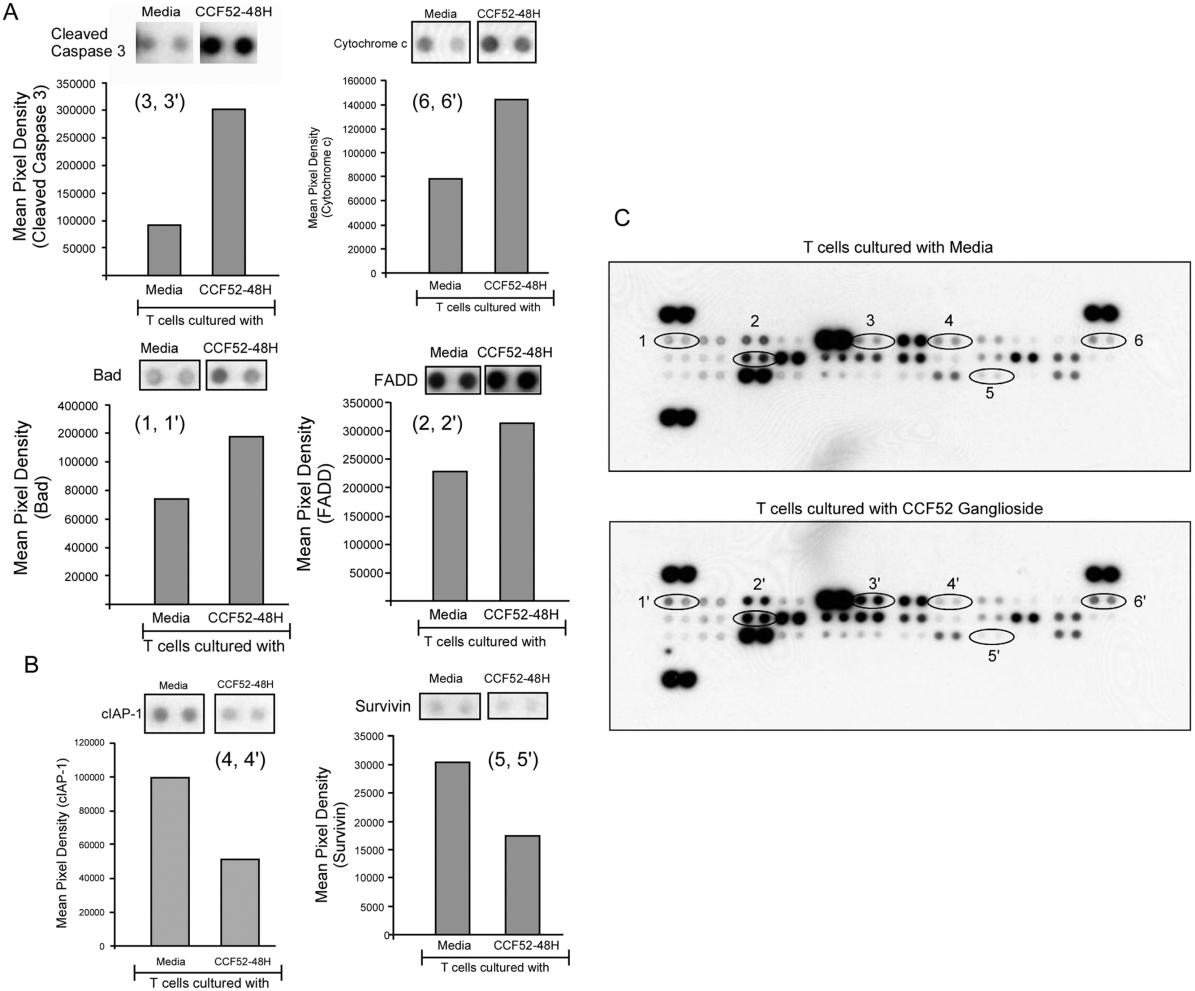
Human apoptosis proteome profiler array demonstrates ganglioside induced activation of pro-apoptotic and downregulation of anti-apoptotic proteins. Differential expression of pro- and anti-apoptotic proteins were examined in T cells cultured with CCF52 ganglioside (15μg/ml) for 48hrs using a human proteome profiler array kit (R&D Biosystems) as represented in Fig 4A and 4B. Fig 4C shows the entire apoptosis proteome profile array of T cells in presence or absence of CCF52 ganglioside. Data is representative of a single experiment out of two experiments done.

To get a global view of differential expression of pro- and anti-apoptotic proteins in T cells in response to GBM gangliosides, normal donor T cells were cultured with CCF52 ganglioside (15μg/ml) for 48hrs. Lysates were used to profile differential expression levels of 56 proteins involved in apoptosis, using a human apoptosis proteome profiler array kit (R&D Systems). Analysis of the membranes indicate significant changes in the expression levels of several different proteins involved in apoptotic machinery, as shown in Fig 4C. As expected caspase-3 and cytochrome-c were found to be upregulated significantly in CCF52 treated T cells versus the control cells (Fig 4A). There were also increased Bad and FADD expression (Fig 4A) while the expression of the anti-apoptotic proteins, cIAP-1 and survivin (Fig 4B) was diminished in T cells incubated with gangliosides. Thus, GBM derived gangliosides promote T cell death not only by inducing pro-apoptotic proteins, but in the process, they decrease the levels of select anti-apoptotic proteins as well.

### Apoptosis induced by GBM derived gangliosides in activated T cells does not involve TNF-α

**Fig 5:**
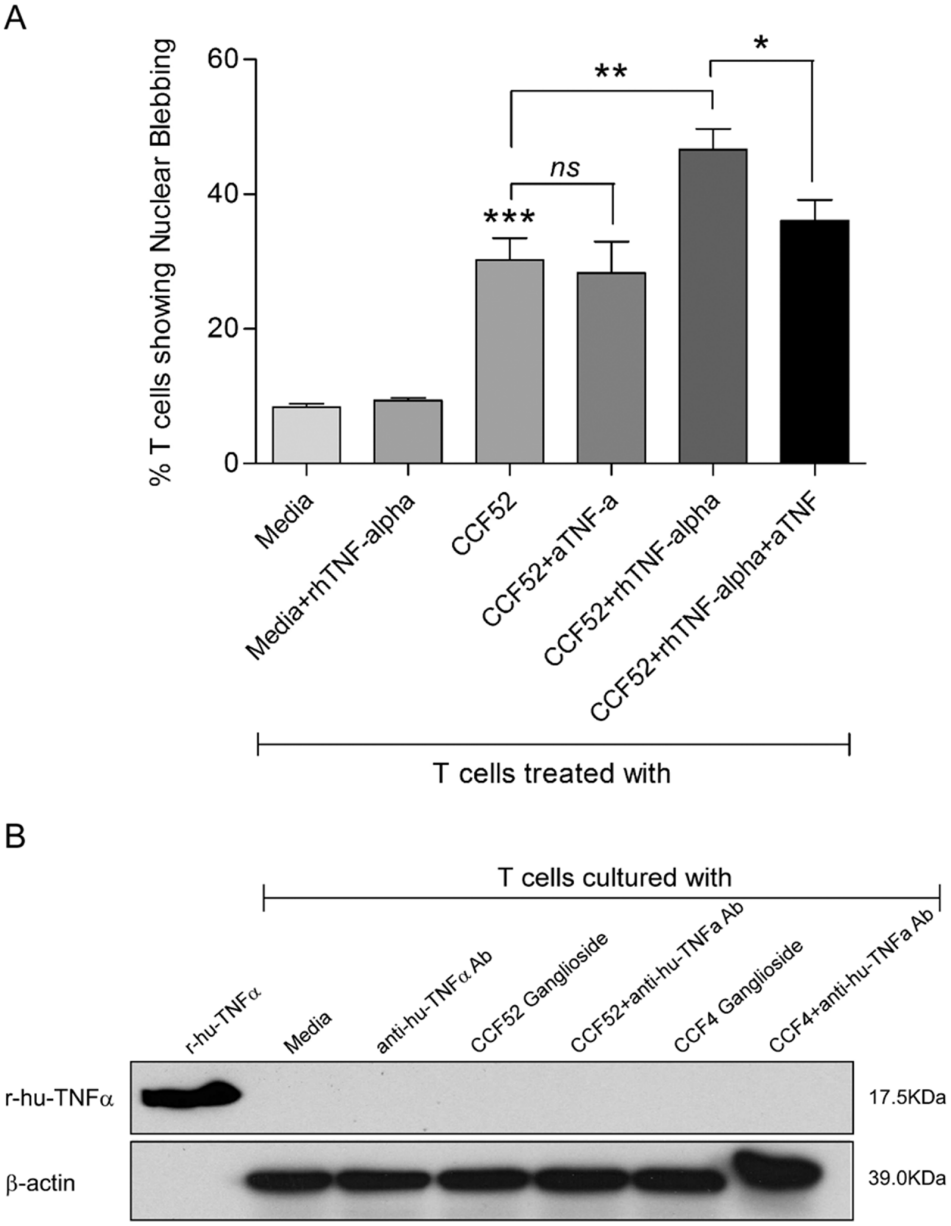
T cell death mediated by GBM derived gangliosides does not involve TNF-α. T cells were treated with anti-human TNFα antibody 2hrs prior to co-culture with either CCF52 ganglioside (15μg/ml) alone or a combination of CCF52 ganglioside and recombinant human TNFα for 72hrs. T cells were then examined microscopically for nuclear blebbing (Fig 5A). While CCF52 ganglioside alone was able to induce significant T cell apoptosis (Mean = 30.21±3.27, ***p<0.001 vs Media), pre-treatment with anti-human TNFα Ab did not offer any significant protection (Mean = 28.30±4.65). Further, while recombinant human TNFα in combination with CCF52 ganglioside showed increased level of T cell apoptosis (Mean = 46.63±3.02, **p<0.01 vs CCF52 ganglioside alone), pre-treatment with anti-TNFα Ab blocked it only to the extent that was induced by exogenously added rhTNFα in combination with CCF52 ganglioside (Mean = 36.07±3.08, *p<0.05 vs CCF52 ganglioside+rhTNFα). Data is representative of at least 3 independent experiments. Data from western immunoblot analysis shows no induction of TNFα in response to CCF52 ganglioside treatment, as evidenced from the absence of any bands corresponding to human TNFα (Fig 5B).

We investigated the role of TNF-α in the induction of GBM derived ganglioside mediated T cell apoptosis. Since, in the tumor microenvironment, tumor infiltrating lymphocytes (TILs) encounter tumor antigens and undergo activation, we thought that it will be physiologically more relevant to study the potential role of TNF-α using 7-day activated T cells (Fig 5). Data shows that while recombinant hTNF-α alone did not induce any significant T cell apoptosis, CCF52 ganglioside alone did induce T cell death (Fig 5A). However, T cell apoptosis induced by CCF52 ganglioside does not likely involve TNF-α since, anti-human TNF-α antibody was unable to block T cell death (Fig 5A). This is similar to the results obtained in a previous report from our laboratory [29], which suggested that GBM cell line induced T cell apoptosis does not involve TNF-α. However, in the presence of TNF-α, T cells show heightened susceptibility to apoptosis (~45%) by GBM derived gangliosides (Fig 5A). This is coherent to the finding from a previous report demonstrating that gangliosides and TNF-α can synergize to induce T cell apoptosis [42]. Addition of TNF-α Ab to the above system blocks T cell death to the extent that was induced by exogenously added rhTNF-α, further ruling out the involvement of any induced endogenous TNF-α in GBM derived ganglioside mediated T cell death. These results indicate that two parallel mechanisms exist for ganglioside mediated T cell death. In the presence of TNF-α, gangliosides synergize with TNF-α to induce T cell apoptosis through involvement of the TNF receptor [42]. However, in the experiments reported here, gangliosides alone mediate T cell apoptosis in the absence of endogenous TNF-α, through involvement of both receptor dependent and receptor independent pathways of caspase activation. As seen in figure Fig 5B, ganglioside treatment did not lead to induction of any TNF-α production in T cells as demonstrated by western blot analysis. Recombinant hTNF-α was used as a positive control.

### GBM derived gangliosides interact directly with TNFR1 leading to downstream recruitment of FADD and TRADD

**Fig 6:**
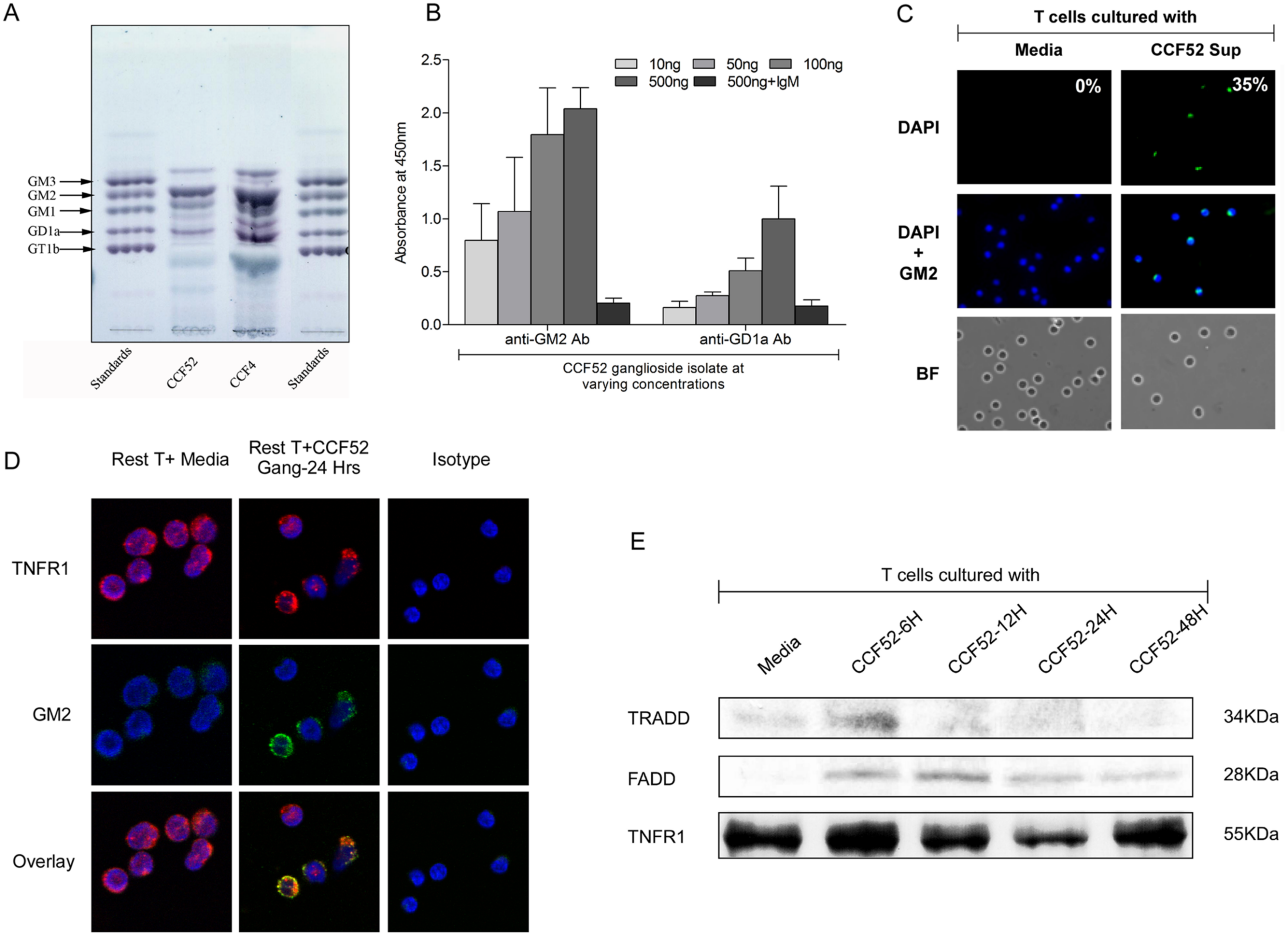
GBM derived gangliosides interact with TNFR1 to induce recruitment of FADD and TRADD leading eventually to downstream activation of caspases. Differential profiles of ganglioside isolates from GBM cell lines CCF52 and CCF4 were obtained by HPTLC using procedure described earlier, as shown in Fig 6A. ELISA assay was also used to detect GM2 and GD1a in extracted gangliosides from CCF52 cell line (Fig 6B). Wells of a 96 well ELISA plate were coated with varying concentrations of CCF52 gangliosides as indicated in the figure legend (Fig 6B). Ganglioside GM2 shed by tumors are taken up by T cells (around 35% GM2-+ve T cells) when cultured with CCF52 supernatants as shown in Fig 6C. Co-localization of GM2 (green) and TNFR1 (red) in T cells treated with CCF52 ganglioside (15μg/ml) for 24hrs was demonstrated from overlay by confocal microscopy (Fig 6D). T cells from normal volunteers were co-cultured with CCF52 gangliosides for 6, 12, 24 and 48hrs. Cells were washed, lysed and co-immunoprecipitated with anti-human TNFR1 Ab. Western immunoblot analysis confirmed that TRADD and FADD co-immunoprecipitated with TNFR1 (Fig 6E), indicating recruitment of the DISC. Representative data from one of 3 experiments is shown.

Since, ganglioside GM2 has been identified as one of the potential candidates for inducing T cell apoptosis mediated by tumor derived ganglioside [22], we used HPTLC to first determine the expression profile of individual gangliosides expressed by CCF52 and CCF4 (Fig 6A). Both of these lines expressed multiple gangliosides including GM2 and GD1a by HTPLC analysis which was confirmed by ELISA (Fig 6B) using hamster anti-human GM2 Ab and mouse anti-human GD1a Ab. When normal T lymphocytes were treated with conditioned media obtained by culturing CCF52 cell lines, ganglioside GM2 was taken up by T cells as evidenced by FITC-positive T cells (Fig 6C), indicating that gangliosides were not only shed by tumor cells but also taken up significantly (~ 35%) by T cells.

**Fig 7:**
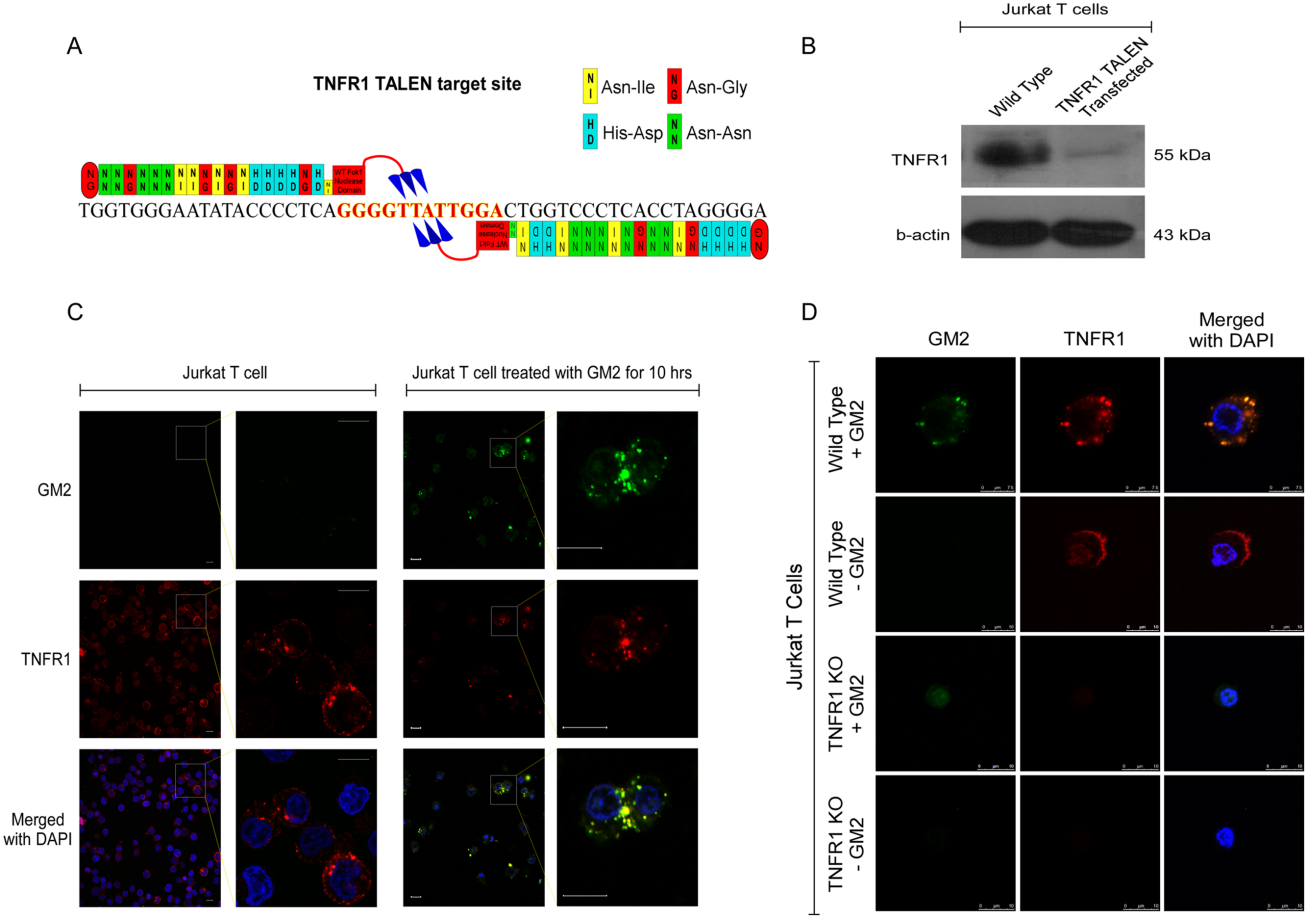
TALEN mediated targeted disruption of TNFR1 gene abolished the ganglioside GM2–TNFR1 interaction in Jurkat-T cells. Fig 7A shows schematic representation of TNFR1 specific TALEN pair. DNA sequence with black letters indicates TALEN target sequence against which TALEN pair has been designed, red letters represent spacer DNA sequences. TALEN modules are represented as yellow, red, green or blue boxes according to their base recognition specificity of A, T, G or C respectively. Large red box with overhanging 3 arrows indicates wild type Fok1 nuclease domain, shown in Fig 7A. Western immunoblotting was performed to check the expression level of TNFR1 transfected, G418 selected Jurkat-T cells vs wild type Jurkat-T cells. β-actin was used as loading control as shown in Fig 7B. Jurkat-T cells treated with GM2 or not for 10hrs were attached in poly-L-lysine coated coverslips and stained with GM2 specific and TNFR1 specific antibodies, counterstained with respective fluorescent tagged secondary antibodies and mounted on slides with Vectashield mounting media containing DAPI and assessed for co-localization of TNFR1 and GM2 under confocal microscope (Fig 7C). Both wild type and TNFR1 KO cells were treated with GM2 of not for 10hrs and processed as described above and visualized under confocal microscope as shown in Fig 7D. Scale bar represents 10μm.

To investigate whether shed gangliosides interact directly with the TNFRI, co-localization studies were performed on T cells incubated with CCF52 derived gangliosides for 24hrs. Immunofluorescent staining with rabbit anti-human TNFRI antibody (Red) and hamster anti-human GM2 antibody (Green) followed by confocal microscopy showed presence of TNFRI in CCF52 treated T cells as well as the control cells (Fig 6D, panel 1) as evidenced from the red fluorescence. GM2 was also shown to be taken up by T cells treated with CCF52 ganglioside at 24hrs, as observed from the green fluorescence in CCF52 treated T cells versus the control cells (Fig 6D, panel 2). An overlay of green and red channels confirm co-localization of GM2 and TNFRI on the T cells treated with CCF52 ganglioside as observed from the orange or yellow fluorescence, characteristic of co-localization (Fig 6D, panel 3). A normal rabbit IgG antibody was used as the isotype control for TNFRI. This co-localization was further validated and confirmed in Jurkat T cell clones (TNFR1 KO), where the exon 2 of TNFR1 gene locus was mutated by targeted genome editing using TALEN technology. TALEN constructs were obtained which were designed in a way to effectively target the TNFR1 exon 2 as shown in Fig 7A. Transfection of Jurkats with left and right TALEN pairs caused targeted editing of the TNFR1 gene, thereby generating TNFR1 KO clone. The TNFR1 clone shows negligible expression of TNFR1 protein expression as validated by western blot analysis shown in Fig 7B. Both wild type and TNFR1 KO Jurkats were then treated or not with exogenous GM2 at time points much earlier than that required for apoptosis induction, to demonstrate co-localization of GM2 with the TNFR1 receptor as shown in Fig 7C and 7D. Data shows co-localization of GM2 (green) with the TNFR1 (red) in wild type Jurkat cells after 10 hrs of GM2 treatment, as evidenced by the yellow fluorescence in Fig 7C. This was further confirmed in another experiment, where co-localization between GM2 and TNFR1 was observed in wild type Jurkat cells versus TNFR1 KO Jurkats, which showed no co-localization because of negligible TNFR1 expression (Fig 7D).

Whether this interaction with TNFRI leads to downstream recruitment of the components of death inducing signaling complex (DISC) namely, FADD and TRADD, was studied by co-immunoprecipitating TNFRI with the individual DISC components, and western immunoblot to probe for FADD and TRADD (Fig 6E). Western blot analysis shows that both TRADD (34KDa) and FADD (28KDa) were co-immunoprecipitated with TNFRI (Fig 6E), suggesting recruitment and activation of the DISC complex. However, TRADD appears to be recruited first (at 6hrs) followed by FADD (at 12hrs) in response to CCF52 ganglioside treatment. TNFRI was used as the loading control in this experiment. These results show that CCF52 ganglioside induced caspase activation is possibly mediated through direct interaction with the TNFRI, followed by recruitment of the individual death domains (FADD and TRADD) of the DISC leading to subsequent activation of caspase-8.

## Discussion

Immune response to human gliomas has been demonstrated [43–45], particularly T cells were found to elicit a response against neoplastic cells in different tissue sites including the central nervous system. However, in spite of a tumor specific immune response and infiltration of immune cells in human brain tumors [46] including lymphocytic infiltrates in CNS tumor microenvironment [47], significant dysfunction of these immune cells is observed [3, 19] likely due to apoptosis of these immune cells [17]. This observed immunosuppression mediated by gliomas poses a barrier to development of an effective immune response to GBM immunotherapy, and hence understanding the underlying mechanisms is key to finding effective strategies in developing a strong immune response.

Previous reports from our laboratory and others have demonstrated the deleterious role of tumor derived soluble products, particularly gangliosides in mediating both RCC [22, 37, 38, 48] and GBM [29] induced immune dysfunction and T cell apoptosis. GBM induced T cell apoptosis is likely mediated through distinct pathways involving gangliosides and CD70 [29]. Our findings in this study reveal how GBM derived gangliosides induce T cell apoptosis (Fig 1) by directly interacting with the TNFRI, thereby recruiting individual components of the death inducing signaling complex (DISC), namely Fas associated death domain (FADD) and TNF receptor associated death domain (TRADD), which in turn activates initiator caspase-8, thereby, activating the receptor dependent pathway of caspase activation.

There are reports which show that TIL T cells are exhausted and dysfunctional, particularly in their ability to elicit an effective anti-tumor response [49, 50]. Interestingly, there are also a large number of reports showing tumor-induced apoptosis of effector T-lymphocytes in different cancers [35, 51, 52]. We believe exhaustion and apoptosis happens in concert and is context dependent. Strong evidences exist which shows elevated levels of gangliosides in T cells (both TILs as well as peripheral blood T cells) from RCC (renal cell carcinoma) patients to be directly associated with T cell apoptosis in RCC patients [37], which proves beyond any doubt that at least in RCC, increased levels of gangliosides in T cells contribute to the heightened apoptosis observed in RCC. Measurement of T cell apoptosis using lower concentrations of CCF52 ganglioside (1, 5 and 10μg/ml) at 48 and 72hrs (Fig 1B) demonstrates a dose- as well as a time-dependent increase in T cell apoptosis induced by CCF52 gangliosides. Although, lower doses, more specifically 1μg/ml and 5μg/ml of CCF52 ganglioside were not sufficient to induce T cell apoptosis either at 48hrs or 72hrs (Fig 1B), however, were effective in blocking T cell function as is evidenced from the dose dependent inhibition of IFN-γ mRNA (S1 Fig).

Since previous reports from our laboratory have suggested the involvement of ganglioside GM2 in mediating T cell apoptosis in RCC [22, 37], confocal microscopy was used to establish whether GM2 co-localizes with TNFRI. Fig 6D clearly demonstrates the possibility of a direct interaction of GM2 and TNFRI. Further confirmation of this interaction was obtained from co-localization studies performed with engineered Jurkat T cells, where the TNFR1 locus was mutated using TALEN assisted targeted editing (Fig 7A and 7B). Although, addition of exogenous GM2 showed positive co-localization with the TNFR1 in wild type Jurkat cells, however, the TNFR1 KO clones showed no co-localization as expected (Fig 7D). Interestingly, there was only a small center of GM2 staining in the TNFR1 KO Jurkats, suggesting that most of the incorporated GM2 primarily binds to TNFR1 but in absence of TNFR1, some GM2 gets internalized but do not co-localize. The outcome of this interaction was further confirmed by co-immunoprecipitation experiments where FADD and TRADD co-immunoprecipitated with TNFRI in T cell lysates treated with CCF52 ganglioside (Fig 6E), thereby indicating time-dependent interaction and formation of DISC in response to ganglioside treatment. This also suggests that tumor derived gangliosides were able to induce receptor dependent T cell death independently, without the involvement of other death inducing ligands like TNF-α. Since, earlier reports have suggested synergy between TNF-α and gangliosides in mediating tumor induced T cell apoptosis [42], our data also presents the possibility of two distinct modes of receptor activation in T cells leading to T cell death, depending on the availability of TNF-α. In the presence of TNF-α, tumor derived gangliosides synergize with the ligand in activating the TNF receptor as suggested previously [42]. However, in our study we used either 7-day activated T cells (activated with anti-CD3/anti-CD28 Ab in presence of IL2) or resting T cells, neither of which express any detectable levels of TNF-α, as shown by western blot analysis (Fig 5B). Further, involvement of TNF-α was ruled out since, anti-human TNF-α Ab was unable to block CCF52 ganglioside mediated T cell apoptosis (Fig 5A). Hence, our data strongly suggests that in absence of TNF-α, tumor derived gangliosides interact directly with the TNFRI, to induce downstream signaling events leading to caspase activation and consequent apoptosis.

Apoptotic signals are amplified by simultaneous mitochondrial involvement through caspase-8 dependent cleavage of Bid to t-Bid, induction of MPT, release of cytochrome c from the mitochondria and activation of caspase-9. Together caspase-8 and caspase-9 activation cause downstream activation of effector caspase-3, which ultimately leads to apoptosis. Involvement of both the receptor dependent and receptor independent modes of caspase activation were demonstrated by time dependent activation of caspases-3, -8 and -9 (Fig 2A, 2B and 2C). This was also confirmed by inhibitor experiments, where specific inhibitors to caspase-3, -8 and -9 as well as the pan caspase inhibitors were able to significantly block CCF52 ganglioside induced T cell death (Fig 2D). Interestingly, all of the caspase inhibitors could only partially block T cell death (% reduction in T cell death in presence of inhibitors is around 50%), suggesting that other modes of cell death (for example autophagy) may also be involved in ganglioside mediated T cell death. Data showing mitochondrial involvement in GBM ganglioside mediated T cell apoptosis indicates both direct damage to the mitochondria through induction of ROS as seen from time dependent increase in MFI values in T cells treated with GBM gangliosides (Fig 3D), and also an indirect way through Bid cleavage (Fig 3A), causing MPT (Fig 3B) and leading to release of cytochrome c from the mitochondria (Fig 3C). This is concurrent with previous reports suggesting mitochondrial involvement in ganglioside mediated T cell death [22, 37, 42, 53].

Finally, human apoptosis proteome array provided a global picture showing changes in expression profiles of several pro- and anti-apoptotic proteins in response to CCF52 ganglioside treatment in T cells (Fig 4C). Data from Fig 4A confirmed caspase-3 activation and also release of cytochrome c, thereby confirming mitochondrial involvement. However, anti-apoptotic proteins like cIAP-1 and survivin were found to be significantly reduced (Fig 4B) in response to ganglioside treatment, indicating that gangliosides not only induce pro-apoptotic signals but also helps suppress anti-apoptotic protein levels thereby further driving the cells towards apoptosis.

In addition to elucidating the precise signaling mechanisms behind GBM ganglioside mediated T cell apoptosis, our studies also revealed the possibility of two distinct pathways of TNF receptor activation. Depending on the cell system and availability of TNF-α, tumor derived gangliosides either synergize with TNF-α to induce T cell death, or, in absence of TNF-α, GBM gangliosides act independently and interact with TNFRI, thereby leading to downstream recruitment of the DISC and activating caspases, eventually leading to T cell apoptosis.

## Supporting Information

S1 Fig GBM derived gangliosides inhibit IFN-γ response in T cells. Purified blood T lymphocytes isolated from blood of healthy volunteers were were co-cultured with varying concentrations of CCF52 and CCF4 gangliosides for 24hrs before stimulating by CD3/CD28 beads for an additional 48hrs for IFN-γ response. Cells were then surface stained for CD3 and intracellular staining for IFN-γ was done. Data shows dose dependent inhibition of intracellular levels of IFN-γ in T cells treated with either CCF52 or CCF4 gangliosides (**p<0.01 Unstimulated vs CD3/CD28 stimulated, **p<0.01 CD3/CD28 stimulated vs CCF52/CCF4 (5μg/ml), *p<0.05 CD3/CD28 stimulated vs CCF52/CCF4 (1μg/ml).
(TIF)
