# Supplementary figures and images for: GBM Derived Gangliosides Induce T Cell Apoptosis through Activation of the Caspase Cascade Involving Both the Extrinsic and the Intrinsic Pathway
Source: PLoS One. 2015 Jul 30;10(7):e0134425. doi: 10.1371/journal.pone.0134425 (PMC4520498; doi:10.1371/journal.pone.0134425)

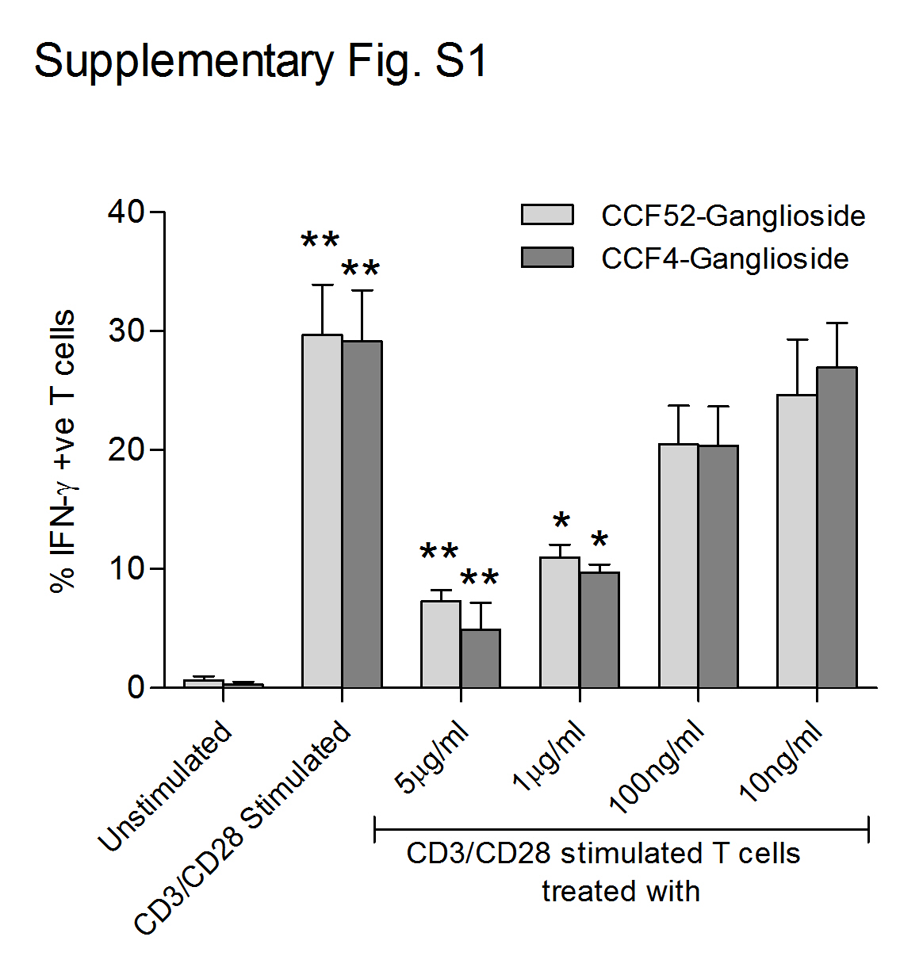

Supplement: S1 Fig — Purified blood T lymphocytes isolated from blood of healthy volunteers were were co-cultured with varying concentrations of CCF52 and CCF4 gangliosides for 24hrs before stimulating by CD3/CD28 beads for an additional 48hrs for IFN-γ response. Cells were then surface stained for CD3 and intracellular staining for IFN-γ was done. Data shows dose dependent inhibition of intracellular levels of IFN-γ in T cells treated with either CCF52 or CCF4 gangliosides (**p<0.01 Unstimulated vs CD3/CD28 stimulated, **p<0.01 CD3/CD28 stimulated vs CCF52/CCF4 (5μg/ml), *p<0.05 CD3/CD28 stimulated vs CCF52/CCF4 (1μg/ml). (TIF) [file pone.0134425.s001.tif]
